# Optimized “In Vitro” Culture Conditions for Human Rheumatoid Arthritis Synovial Fibroblasts

**DOI:** 10.1155/2014/702057

**Published:** 2014-11-04

**Authors:** Claudia Casnici, Donatella Lattuada, Noemi Tonna, Katia Crotta, Claudio Storini, Fabio Bianco, Marcello Claudio Truzzi, Costantino Corradini, Ornella Marelli

**Affiliations:** ^1^Department of Medical Biotechnologies and Translational Medicine (BIOMETRA), University of Milan, Via Vanvitelli 32, 20129 Milan, Italy; ^2^Neuro-Zone s.r.l, Viale Ortles 22/4, 20139 Milan, Italy; ^3^Orthopaedic Hospital Gaetano Pini, Piazza A. Ferrari 1, 20122 Milan, Italy; ^4^Sanipedia s.r.l, Via Podgora 7, 20122 Milan, Italy; ^5^Centro Studi e Ricerche in Traumatologia dello Sport, University of Milan c/o I Divisione, Istituto Ortopedico G. Pini, Piazza A. Ferrari 1, 20122 Milan, Italy

## Abstract

The composition of synovial fluid in rheumatoid arthritis (RA) is complex and strongly influences the microenvironment of joints and it is an inseparable element of the disease. Currently, “in vitro” studies are performed on RA cells cultured in the presence of either recombinant proinflammatory cytokines-conditioned medium or medium alone. In this study, we evaluated the use of synovial fluid, derived from RA patients, as optimal culture condition to perform “in vitro” studies on RA synovial fibroblasts. We observed that synovial fluid is more effective in inducing cell proliferation with respect to TNF-alpha or culture medium alone. Spontaneous apoptosis in fibroblasts was also decreased in response to synovial fluid. The expression of proinflammatory cytokines in the presence of synovial fluid was significantly elevated with respect to cells cultured with TNF-alpha or medium, and the overall morphology of cells was also modified. In addition, modulation of intracellular calcium dynamics elicited in response to synovial fluid or TNF-alpha exposure is different and suggests a role for the purinergic signalling in the modulation of the effects. These results emphasize the importance of using RA synovial fluid in “in vitro” studies involving RA cells, in order to reproduce faithfully the physiopathological environmental characteristic of RA joints.

## 1. Introduction

Rheumatoid arthritis (RA) is an inflammatory disease characterized by infiltrating leukocytes, monocytes, and macrophages in the joint synovial fluid (SF) and in the surrounding synovial tissue [1]. These cells play a critical role in the pathogenesis of RA, by secreting proinflammatory cytokines, which, in turn, perpetuate joint destruction [2, 3]. Fibroblast-like synoviocytes (FLS), in the synovial intimal lining, also contribute to inflammation of the joint and destruction of the cartilage and bone, through cell-cell contact as well as elaboration of soluble products, such as cytokines, chemokines, growth factors, prostaglandins, and leukotrienes [4]. SF thus condenses a mix of factors, secreted by all the cells located within the joint, with prosurvival and proliferative effects that are important in the development of expanded fibroblast network and in the maintaining the inflammatory response [4, 5].

Synovial fluid from RA patients (RA SF) contains higher levels of IL-1*β*, IL-6, and TNF*α* than synovial fluid from osteoarthritis (OA) patients [6–8]. The effects of these cytokines include the induction of cytokine synthesis, the upregulation of adhesion molecules, the activation of osteoclasts, the induction of other inflammatory mediators such as prostaglandins, nitric oxide, and matrix metalloproteinases, the induction of the acute phase response, and the activation of B cells (IL-6).

The RA joint microenvironment also contains high levels of IL-15, it stimulates migration of neutrophils and T lymphocytes into the joint [9, 10], it protects these cells from apoptosis [11], and it induces triggering and proliferation of T cells [12, 13]. In addition, IL15 indirectly induces the expression of proinflammatory cytokines TNF-*α*, IL-1*β*, IL-17, and IL-8, as well as inflammation-inciting free radicals [14–18]. IL15 also participates in FLS activation [10] and in the local bone destruction [19].

IL-17 is higher in RA SF, compared with the concentrations found in OA patients [20]. This cytokine has pleiotropic effects on leukocytes and stromal cells, and it induces IL-6 and IL-8 production by fibroblasts and stimulates macrophage IL-1 and TNF-*α* production [21]. IL-17 is thought to play a crucial role in the bone reabsorption process during RA [21]. Other cytokines increased in RA SF are as follows: IL-8 [22] involved in cellular recruitment, GM-CSF involved in macrophage development, and IL-23 involved in increasing Th17 lymphocytes differentiation [5]. In RA SF, there are also soluble mediators of inflammation, such as prostaglandins, leukotrienes, and matrix metalloproteinases, which may diffuse from blood and/or be formed locally within the joint cavity. Elevated IL-18 levels have been detected in synovial tissues and synovial fluids of RA patients, in significantly higher levels with respect to OA controls [23]. This inflammatory cytokine acts as an angiogenic mediator and leukocyte chemoattractant [24].

RA synovial cells are characterized by a resistance to apoptosis, and this contributes to the accumulation of cells in the rheumatoid lesion. In the inflamed rheumatoid joint, synovial fluid factors tend to inhibit the apoptosis of CD4^+^ T cells, neutrophils [25, 26], and FLS [4]. In addition, this impaired apoptosis may contribute to the development of the microenvironment and to maintain the inflammatory response that characterizes established RA [4, 27]. SF factors that contribute to cells survival are the high levels of common *γ*-chain cytokines (IL-2, IL-4, and IL-15), G-CSF, GM-CSF, and macrophage derived IFN-*α*. Nitric oxide (NO) levels are also increased in RA synovial fluid. NO mediates many different cell functions at the site of synovial inflammation, including cytokine production, signal transduction, mitochondrial functions, and apoptosis [28, 29].

Multiple mechanisms by which NO may regulate apoptosis have been identified [30], one of them might be the suppression by NO donor of the proteolytic processing and activation of caspase-3 in Fas-treated synovial cells [31]. Like NO, thioredoxin 1 (Trx1) levels are elevated in the synovial tissue, fluid, and serum of RA patients [32, 33]. Trx1 has multiple biological activities in regulation of apoptosis, albeit the mechanisms responsible for these activities have not been completely established [29].

The composition of SF is very complex and strongly influences the microenvironment of joints, thus representing an inseparable element of the disease. For these reasons we think that “in vitro” studies about the RA pathology should be performed in SF conditioned medium, rather than recombinant cytokines conditioned medium or medium alone, in order to recreate the correct physiopathological microenvironment, typical of RA. In our study we compared the effect of SF, TNF*α*, and the medium alone on “in vitro” RA FLS, which play pivotal roles both in the initiation and the perpetuation of RA. We observed a greater response of the synovial fibroblasts to SF to confirm our hypothesis.

## 2. Material and Methods

### 2.1. Patients

Synovial membranes were obtained from 10 RA patients undergoing synovectomy or arthroplasty. SF was obtained from 30 patients with established RA. The study was approved by the Hospital Ethics Committee of the Gaetano Pini Hospital of Milan (Italy). All patients signed informed consent to take part in the study.

### 2.2. Isolation of Synovial Fibroblasts and Cell Cultures

Primary RA synovial fibroblasts were isolated from the synovial tissue of RA patients. The tissues were minced and treated for 4 h with 2.5 mg/mL of type I collagenase (Sigma Aldrich, Saint Louis, MO, USA) in Dulbecco's modified Eagle's medium (DMEM) (Euroclone, Italy) at 37°C in 5% CO_2_. Dissociated cells were then centrifuged at 1000 ×g, resuspended in DMEM supplemented with 10% fetal calf serum (FCS) (Fetalclone1 Hyclone Logan, UT, USA), 2 mM L-glutamine, 100 units/mL penicillin, and 100 mg/mL streptomycin (Euroclone, Italy), and plated in 75 cm^2^ flasks (Primo Cell Culture Flask, Euroclone, Italy). After overnight culture, nonadherent cells were removed, and adherent cells were cultivated in DMEM supplemented with 10% FCS. The cultures were kept at 37°C in 5% CO_2_, and the medium was replaced every 3 days. The purity of the cells was tested by flow-cytometric analysis using phycoerythrin-conjugated anti-CD14 (PharMingen, San Diego, CA, USA) and fluorescein isothiocyanate phycoerythrin-conjugated anti-CD3, anti-CD19, anti-CD14, or anti-Thy-1 (CD90) monoclonal antibodies (R&D Systems Minneapolis, MN). A FACS Calibur flow cytometer (488Ex/620Em) (Becton Dickinson, San José, CA, USA) was used for the analysis. At passage 3, the cells were morphologically homogeneous and exhibited the appearance of FLS, with typical bipolar configuration under inverse microscopy. Most cells (>98%) expressed the surface markers for fibroblasts (Thy-1) and were negative for the expression of CD3, CD19, and CD14. Synoviocytes from passages 3–8 were used in each experiment.

SF was directly aspirated from the joints of RA patients, and the fluid was collected into heparinized tubes and spun at 1000 ×g for 10 min. The acellular portion of SF was stored at −80°C before use.

### 2.3. Synovial Fibroblasts Proliferation Assays

FLS were cultured in complete medium alone or in the presence of recombinant human tumor necrosis factor-alpha (TNF*α*) (25 ng/mL) (Immunological Sciences, Italy) or SF, at the dilution of 1 : 8 in culture medium. We used three pools of SF, each from 10 RA patients' fluid, in order to minimize the variability in responses among different pools. At indicated time points the evaluation of newly synthesized DNA was carried out using the Click-iT EdU HCS Assay (Life Technologies Italia) in accordance with the kit instructions. 5-Ethynyl-2′-deoxyuridine (EdU) (10 *μ*M) was added to the cultures 24 h before the detection, cells were then fixed and permeabilized, and EdU incorporated into newly synthesized DNA was detected using the fluorescent Alexa Fluor 488 azide. Analysis was performed using a confocal microscopy. Data were obtained from triplicate experiments.

### 2.4. Detection of Apoptosis

The detection of apoptosis was performed by the annexin V-fluorescein isothiocyanate (FITC)/propidium iodide (PI) kit (BioLegend, San Diego, CA) according to the manufacturer's instructions. Briefly, after treatments cells were washed and resuspended in annexin V binding buffer, and then annexin V-FITC and propidium iodide (PI) solution were added. After 15 min at room temperature in the dark, annexin V binding buffer was added. The resulting fluorescence was detected by flow cytometry (FACScan-Becton Dickinson, Mountain View, CA) with CellQuest analysis software. At least 2000 cells were analysed for each sample. The percentage of apoptosis was calculated as apoptotic index considering cells both in early and late apoptosis.

### 2.5. Real-Time RT-PCR

FLS were incubated in absence or presence of TNF*α* (25 ng/mL) or SF (1 : 8 dilution in culture medium). After 7 days cells were processed in triplicate with the TaqMan Gene Expression Cells-to-CT Kit (Life Technologies Italia) according to the manufacturer's instructions. Briefly, cells were washed with cold PBS and resuspended in Lysis Solution + DNAse I and incubated for 5 min at room temperature. Stop solution was then added and after 2 min of incubation at room temperature the samples were transferred at −80°C. TaqMan Gene Expression Master Mix and TaqMan Gene Expression Assays (Life Technologies Italia) were used to perform real-time quantitative polymerase chain reaction. mRNA expression of IL1*β*, IL6, IL15, and TNF*α* was evaluated. Data were analysed according to the comparative Ct method and were normalized by GAPDH expression in each sample.

### 2.6. Actin Cytoskeleton

FLS were cultured on coverslips to a 10% to 20% confluence and then incubated in absence or presence of TNF*α* (25 ng/mL) or SF (1 : 8 dilution in culture medium). After 7 days cells were fixed with 4% formaldehyde for 15 min at room temperature followed by permeabilization of the cells with PBS plus 0.1% Triton X-100 for 5 min. 5% nonfat milk (30 min) was used to prevent nonspecific binding. Cells were incubated with Alexa Fluor 594 Phalloidin (actin filament staining; Invitrogen) for 15 min. The stained cells were washed with PBS and mounted on a glass slide. A Zeiss Axiovert 200M fluorescent microscope was used for visualization with the appropriate filters, with Zeiss Axioversion 4.7 software (Jena, Germany).

### 2.7. Intracellular Calcium Measurements

Cultures were loaded for 35–40 min at 37°C with 2 *μ*M Fura-2-AM in Krebs-Ringer solution buffered with HEPES, 125 mM NaCl, 5 mM KCl, 1.2 mM MgSO_4_, 2 mM CaCl_2_, 10 mM glucose, and 25 mM HEPES (pH 7.4) and were washed twice with prewarmed Krebs-Ringer solution before recordings were made. The recording setting comprised an inverted microscope (Leica, DMI600B) equipped with a Ca^2+^ imaging unit. Polychrome IV (TILL Photonics; Germany) was used as a light source. Fura-2 fluorescence images were collected with a Andor CCD Camera (Axon Instruments, CA, USA) and analyzed with Imaging WorkBench 6 (INDEC BioSystem, Santa Clara USA). Single-cell 340/380 nm fluorescence ratios were analysed with Origin 6.0 (Microcal Software Inc., MA, USA).

### 2.8. Statistical Analysis

Statistical analysis was performed using Student's *t*-test for matched pairs. Differences with a confidence level of >95% were considered statistically significant (*P* < 0.05).

## 3. Results

To compare the effect of SF, TNF*α*, and culture medium alone, we first evaluated the proliferation of synovial fibroblasts, established from rheumatoid synovial tissue. Cells were cultured in medium additioned with 10% FCS in the presence/absence of either TNF*α* or SF from RA patients. We utilized TNF*α* at the optimal concentration of 25 ng/mL as our reference standard, since this concentration is known to induce maximal cell proliferation of synovial fibroblasts. We used three SF pools; at a final dilution of 1 : 8 in culture medium, the lower dilution is able to induce maximal cell proliferation of synovial fibroblasts (data not shown).

**Figure 1:**
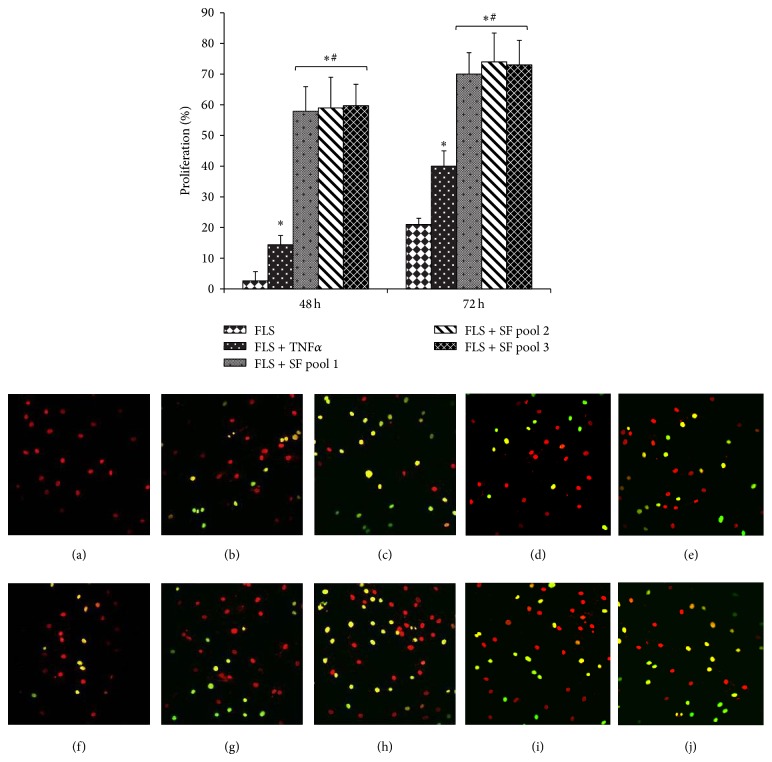
RA fibroblast-like synovial cells proliferation in the presence of synovial fluid or TNF*α*. FLS were cultured in the presence of TNF*α* (25 ng/mL) or three SF pools (1 : 8 dilution in culture medium). The DNA synthesis was evaluated after 24 h incubation with 10 *μ*M 5-ethynyl-2′-deoxyuridine (EdU). Cells were then fixed and permeabilized, and EdU incorporated into newly synthesized DNA was detected using the fluorescent Alexa Fluor 488 azide. Analysis was performed using a confocal microscope. Data were obtained from triplicate experiments. Results are shown as % of proliferation: % = (nuclei stained with Edu/total nuclei) × 100 and represent four independent experiments. Confocal microscopy images of a representative experiment are shown: cells cultured for 48 h in alone medium (a), TNF*α* (b), or SF pools (c, d, e), cells cultured for 72 h in medium alone (f), TNF*α* (g), or SF pools (h, i, j). Green staining: newly synthesized DNA, red staining: Hoechst staining of total DNA. ^*^ *P* < 0.05 versus FLS; ^#^ *P* < 0.05 versus FLS + TNF*α*.

**Figure 2:**
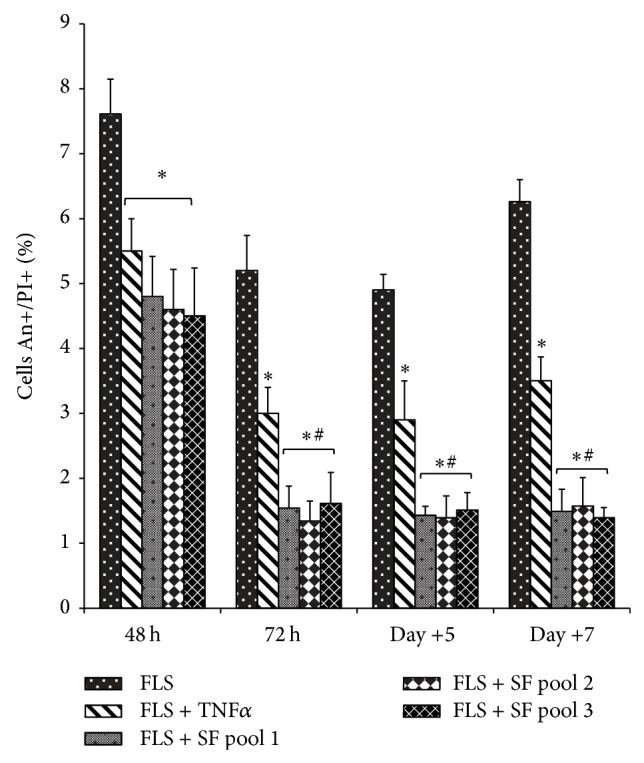
Effects of synovial fluid and TNF*α* on spontaneous apoptosis of RA fibroblast-like synovial cells. RA synovial fibroblasts were cultured in the presence of TNF*α* (25 ng/mL), three SF pools (1 : 8 dilution in culture medium), or medium alone. Apoptosis was evaluated at the indicated times. Cells were stained with annexin V-FITC and propidium iodide and analysed by flow cytometry. Apoptotic cells were measured as the percentage of annexin V-positive/PI-positive cells. Results are expressed as the mean percentage ± SD from four individual experiments. ^*^ *P* < 0.05 versus FLS; ^#^ *P* < 0.05 versus FLS + TNF*α*.

Exposure to SF provided a significant growth advantage for FLS, compared with exposure to TNF*α* or medium alone, with an increase in DNA synthesis that was evident already after 48 h of incubation, and it was also maintained at later times (Figure 1). Comparing the three different pools, we did not observed a significant difference in cellular responses. We also evaluated the influence of SF on apoptosis, given that it is known that RA FLS are more resistant to apoptosis, and one of the possible mechanisms responsible for this resistance is the presence of antiapoptotic factors in the SF. We assessed the spontaneous apoptosis of FLS cultured in medium alone, in medium with SF or with TNF*α*. Our results indicate that the presence of SF in the culture medium significantly decreased apoptosis, compared to the medium alone, and this protective effect on the cells increased when we extended the treatment beyond 48 h (i.e., 7 days). Also in this condition, no differences between the three SF pools were observed. In the presence of TNF*α*, a protective effect on apoptosis was observed, but this effect was less than the one induced by SF (Figure 2).

**Figure 3:**
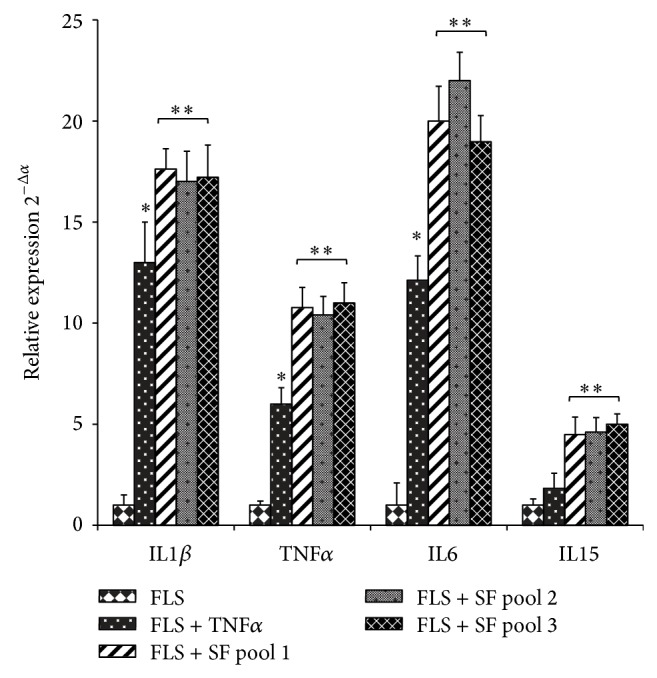
Effects of synovial fluid and TNF*α* on mRNA expression of proinflammatory cytokines in RA fibroblast-like synovial cells. FLS were incubated in absence (FLS) or presence of TNF*α* (25 ng/mL) (FLS + TNF) or three different synovial fluid pools (FLS + SF) (1 : 8 dilution in culture medium). After 7 days cells were analysed for IL1*β*, IL6, IL15, and TNF*α* mRNA expression by quantitative RT-PCR. Data were analysed according to the comparative Ct method and were normalized by GAPDH expression in each sample. Relative mRNA levels were calculated based on the Ct values, corrected for GAPDH expression, according to the equation: 2^−ΔCt^ [ΔCt = Ct(TNF*α*, IL1*β*, IL6, or IL15) − Ct(GAPDH)]. Data are expressed as mean ± SD. ^*^ *P* < 0.05 versus FLS; ^**^ *P* < 0.05 versus FLS + TNF*α*.

**Figure 4:**
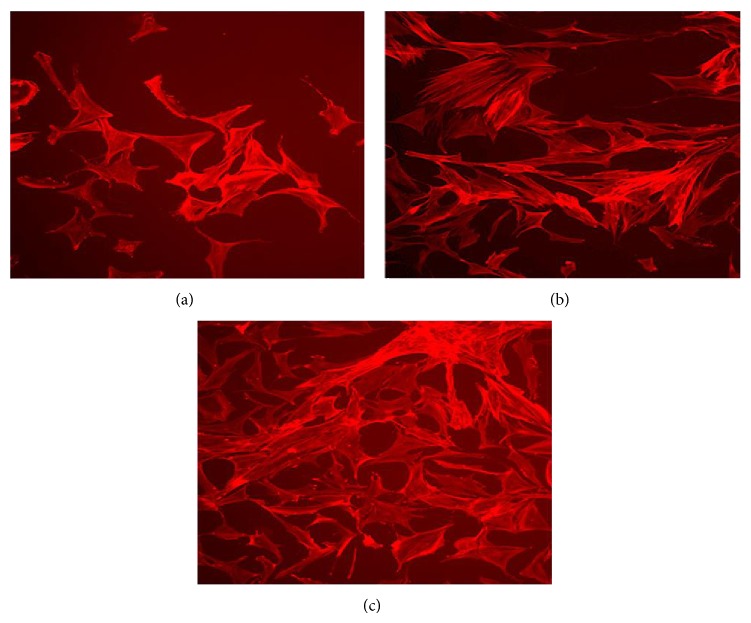
Effect of synovial fluid and TNF*α* on cytoskeleton reorganization in fibroblast-like synovial cells. RA synovial fibroblasts were cultured for 7 days in the presence of TNF*α* (25 ng/mL) (b), SF pool 1 (1 : 8 dilution in culture medium) (c), or medium alone (a). Actin cells were stained with Alexa Fluor 594-Phalloidin.

Then, we explored the modulation of the expression of four inflammatory cytokines known to be involved in RA pathology, namely, IL1*β*, IL6, IL15, and TNF*α*. FLS were exposed to SF or TNF*α* and selected gene expression was then quantified by real-time PCR. As shown in Figure 3, unstimulated FLS constitutively produced IL1*β*, IL6, IL15, and TNF*α*, as expected, and the chronic exposure to SF or TNF*α* increased the expression of these cytokines. The mRNA expression was strongest in cells exposed to the three SF pools, and its maximal stimulatory effect was detected after 7 days of exposure. Given that we did not evidence significant differences of the responses interpool, neither in cell proliferation and apoptosis nor in the expression of cytokines, we used a single SF pool for the subsequent experiments. We concentrated our attention on the effect of SF and TNF*α* on cytoskeletal reorganization in RA FLS (Figure 4). Indeed, it is known that these cells exhibit rearranged cytoskeleton that may lead to morphological changes, ultimately resulting in the “transformed” phenotype of the cells, characterized by increased migration, adhesion, and proliferation properties. We observed that “in vitro” treatments with SF and TNF*α* induced the reorganization of the actin cytoskeleton, but SF effects on cytoskeleton and morphological changes were more pronounced, with an observable cell spreading effect.

**Figure 5:**
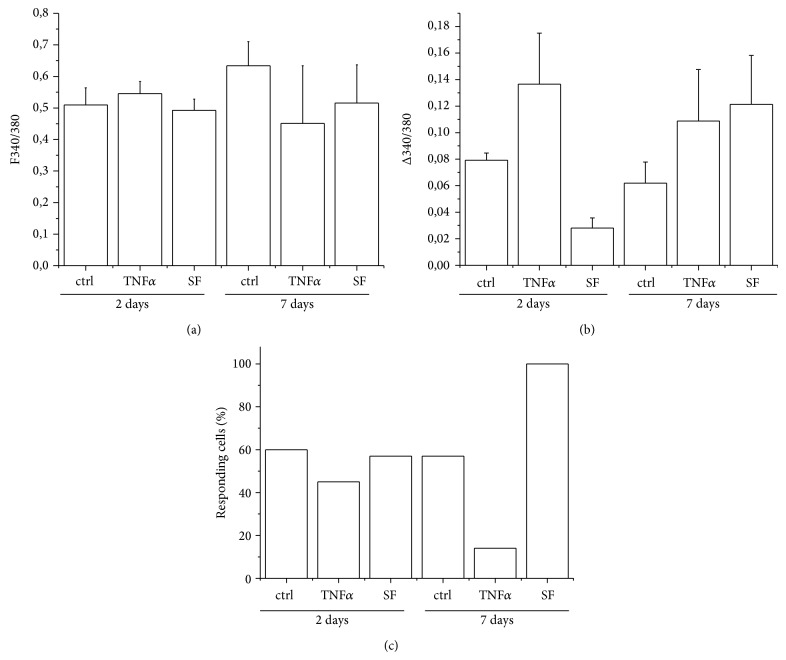
Effect of TNF*α* and synovial fluid on intracellular calcium dynamics in ATP-challenged fibroblast-like synovial cells. (a) Quantitative evaluation of basal intracellular calcium levels in FLS exposed to either TNF*α* or SF pool 1 for either 2 or 7 days. No significant changes in basal intracellular calcium dynamics in the different experimental conditions are observed. A drastic change in amplitude of response (b) and percentage of responding cells (c) to 1 mM ATP is observed between TNF*α* and SF samples. In particular, TNF*α* seems to have an acute effect at shorter times of exposure on the quantity of functional P2 receptor at the plasma membrane, while SF samples show an important change in responsiveness at longer times of exposure.

Subsequently, a functional characterization of FLS, exposed to either TNF*α* or SF, was carried out by quantitative evaluation of intracellular calcium dynamics. As a first step, RA fibroblasts were exposed to TNF*α* or SF for either 2 or 7 days prior to experimental recording. A quantitative evaluation of basal intracellular calcium levels did not show particular changes in the resting status of FLS exposed to the different conditions (Figure 5(a)). However, when the same cells were exposed to inflammatory challenge (such as 1 mM ATP resembling exacerbated extracellular inflammatory conditions, such as the ones which characterize RA pathological contexts), significant differences were observed, both in terms of percentage of responding cells as well as peak intensity of response. In particular, already after 48 h of exposure, FLS primed with TNF*α* showed a significantly higher intensity of response with respect to SF (Figure 5(b)), even though this was not coupled to a concomitant change in percentage of responding cells (Figure 5(c)), thus suggesting an acute effect of TNF*α* on the presence of P2 purinergic receptors at the plasma membrane. At 7 days, the situation was somewhat even more drastic but different, with a significantly higher increase both in the percentage of responsive cells as well as in the mean peak amplitude of response in SF-primed FLS, as compared with TNF*α* samples (Figures 5(b) and 5(c)).

**Figure 6:**
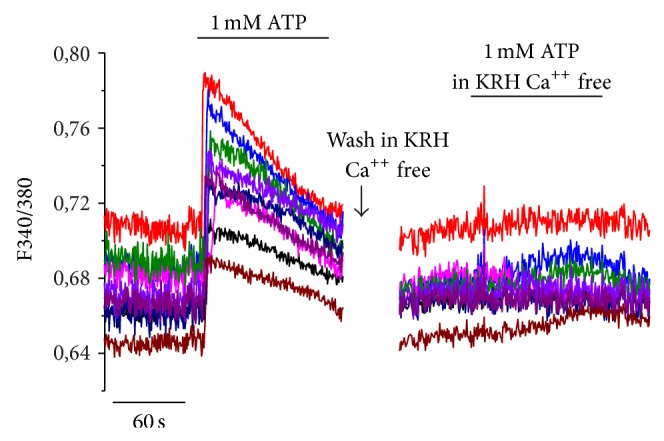
P2X but not P2Y-dependent response of fibroblast-like synovial cells exposed to synovial fluid. Representative graph showing kinetics of response of FLS exposed to SF pool 1 for 7 days. It is very interesting to observe that after 7 days, all RA FLS exposed chronically to SF respond to ATP exposure. When the experiment is replicated in the absence of extracellular calcium, the functional response is completely abolished. This suggests that the ATP mediated response of FLS is due to P2X and not P2Y receptor.

Overall, TNF*α* exposure seemed to have an acute effect at shorter times of exposure on the quantity of functional P2 receptor at the plasma membrane of FLS, which is somewhat maintained at longer times of exposure; on the contrary, SF-exposed FLS showed an important change in responsiveness at longer times of exposure. It is very interesting to observe that after 7 days, all RA FLS exposed chronically to SF responded to ATP exposure with a rapid kinetic, typical of ionotropic channel activation. In order to confirm this hypothesis, the experiment was replicated by administering 1 mM ATP in the absence of extracellular calcium. FLS functional response was in this case completely abolished (Figure 6). These data suggest that the ATP-mediated response of FLS is due to the ionotropic component of the purinergic receptor family (P2X), rather than the metabotropic component (P2Y receptor).

## 4. Discussion

The composition of SF in RA pathology is very complex and strongly influences the microenvironment of joints. In fact, it is an inseparable element of the disease and for these reasons we think that “in vitro” RA studies should be performed in the presence of SF in the culture medium, to recreate the physiopathological microenvironment of RA. In this study, we compared the effect of TNF*α*, a proinflammatory cytokine typically used for “in vitro” RA studies, with respect to SF and the culture medium alone on RA fibroblast synovial cells, to verify our hypothesis. Our observations were performed using three different pools of SF, each from 10 RA patients' fluid. We used the pools of RA synovial fluids to minimize the variability in responses between the different individual synovial fluid samples and to ensure the reproducibility of the results. In addition, we decided to use pools composed of 10 RA patients' fluid to reduce the variability in responses between the different pools. We used FLS because their involvement and critical contribution to the initiation and perpetuation of the disease are well known and are characterized, along with their ability to modulate both joint destruction and propagation of inflammation [4]. Challenged FLS show alterations in morphology and behaviour, including apoptotic responses, inappropriate production of chemokines, adhesion molecules, and matrix-degrading components. For their active role in RA, FLS represent today an important target for novel therapeutic approaches aimed at inhibiting joint destruction. It is well known that the significantly increased proliferation and insufficient apoptosis of FLS are implicated in the pathogenesis and progression of RA [34, 35]. Our observations indicate that the presence of SF in the culture medium can strongly influence these aspects of FLS, in a more efficient way than that observed in the presence of TNF*α* and culture medium alone (Figures 1 and 2). Indeed, all the three SF pools provided a significant growth advantage for FLS, their effect on DNA synthesis was more evident, compared with TNF*α* or medium alone, and it was also maintained for longer time in culture (data not shown). Along the same line, the spontaneous apoptosis of FLS observed was decreased in the presence of the SF pools. This effect was detected starting from 48 h following treatment, with a more pronounced effect at 72 h, and the persistence of SF for longer times in the culture medium stabilized apoptosis. After 7 days, an increase in spontaneous apoptosis in cells cultured with medium alone was detected, probably due to nutrient limitations in the culture supernatant and/or the accumulation of metabolites, while the cells cultured in the presence of SF maintained a very low level of apoptosis, significantly lower than TNF*α*-cultured cells. Here we did not want to analyse the mechanisms which promote resistance to apoptosis in SF-maintained synovial cells, given that this aspect is documented and many factors contribute to this mechanism [27–29, 31], but our observations highlight the important role of SF in conferring this resistance to RA FLS “in vitro.” For this reason, we think that SF should be used as a more physiologically relevant tool in “in vitro” studies, in particular when new therapeutic molecules involved in apoptotic pathways are evaluated.

When we compared the effects of SF and TNF*α* on the expression of cytokines known to be involved in RA pathology, such as IL1*β*, IL6, IL15, and TNF*α*, we observed that chronic exposure to SF (7 days) induced more pronounced effects on the cytokines synthesis in synovial fibroblasts (Figure 3). This supports our hypothesis that the presence of SF would ensure “in vitro” FLS culture conditions more similar to the real environmental characteristics of the RA joints. SF influences the expression of genes involved in modulation of synovial cells and these cells have specific characteristics due to their high inflammation state. In respect to the three different SF pools, we did not evidence significant differences of the responses interpool, neither in cell proliferation and apoptosis nor in the expression of cytokines (Figures 1, 2, and 3), to confirm that the elevated number of synovial fluid samples, 10, used in each pool reduces the variability in responses between the different pools. This is of particular importance when considering the possibility of using such a pool for drug profiling purposes.

Cytoskeleton plays a critical role in the regulation of various cellular processes linked to cell transforming and tumorigenesis, such as contact inhibition and anchorage-independent cell growth [36]. RA FLS exhibit rearranged cytoskeleton, and this reorganization may lead to morphological changes in the FLS, ultimately resulting in the “transformed” phenotype of these cells, characterized by increased migration, adhesion, and proliferation properties [37].

Previously reports found that recombinant TNF*α* induced morphological changes [38] as well as the reorganization of the actin cytoskeleton. In addition, it was demonstrated that pharmacological inhibition of actin cytoskeleton dynamics alters potential pathogenic properties of the synovial fibroblast, such as proliferation, migration, and resistance to apoptosis. Here we compared the effect of TNF*α* and SF on cytoskeleton reorganization (Figure 4) and observed that although both “in vitro” treatments induced the reorganization of the actin cytoskeleton, FLS cultured with SF exhibited more pronounced stress fibres and more significant effects on cytoskeletal and overall morphological changes.

When we focused our attention on the functional responses of FLS subjected to the different experimental scenarios, in terms of quantitative evaluation of intracellular calcium dynamics, we confirmed a clear distinct effect of TNF*α* and SF on FLS responsiveness. In particular, TNF*α* exerts a very rapid effect, with an increase in the peak of the intensity response to 1mM ATP exposure. This effect is maintained at longer times. In contrast, FLS exposed to SF show a significant change at longer times of stimulation (i.e., 7 days) with a striking increase in the percentage of responding cells as well as in the mean peak amplitude of response, thus suggesting a more chronic effect on the purinergic receptor portfolio at the plasma membrane surface of challenged FLS. We tried to give hints on the purinergic receptor components influencing such differences and observed that FLS response was completely abolished when cells were challenged with ATP in the absence of extracellular calcium, thus suggesting a role for the P2X rather than the P2Y component of the purinergic receptor family in the observed effects.

A complete characterization of the purinergic receptor components on the surface of FLS, challenged with either TNF*α* or SF, would be worthy of future investigation in order to give insights on the molecular reasons behind this clearly observed effect.

## 5. Conclusion

These results emphasize the importance of using the RA SF in “in vitro” studies of RA synovial fibroblasts rather than typically used TNF*α* challenge, in order to ensure culture conditions as similar as possible to those typical of the physiopathological environmental in rheumatoid arthritis joints. This should ensure the possibility to have more accurate biological information, both to clarify the pathogenesis of rheumatoid arthritis and to evaluate the therapeutic effectiveness of new molecules.
